# Genetic Analysis in Fetal Skeletal Dysplasias by Trio Whole-Exome Sequencing

**DOI:** 10.1155/2019/2492590

**Published:** 2019-05-14

**Authors:** Kai Yang, Ming Shen, Yousheng Yan, Ya Tan, Jing Zhang, Jue Wu, Guangming Yang, Shang Li, Jing Wang, Zhuo Ren, Zhe Dong, Shan Wang, Manli Zhang, Yaping Tian

**Affiliations:** ^1^Laboratory of Translational Medicine, Beijing Key Laboratory of Chronic Heart-Failure Precision Medicine, Chinese PLA General Hospital, Beijing 100853, China; ^2^Department of Obstetrics and Gynecology, Peking University International Hospital, Beijing 102206, China; ^3^Prenatal Diagnosis Center, Shijiazhuang Obstetrics and Gynecology Hospital, Shijiazhuang, Hebei 050011, China; ^4^Department of Anesthesiology and Operating Room, Peking University People's Hospital, Beijing 100044, China

## Abstract

Skeletal dysplasias (SDs) comprise a series of severe congenital disorders that have strong clinical heterogeneity and usually attribute to diverse genetic variations. The pathogenesis of more than half of SDs remains unclear. Additionally, the clinical manifestations of fetal SDs are ambiguous, which poses a big challenge for accurate diagnosis. In this study, eight unrelated families with fetal SD were recruited and subjected to sequential tests including chromosomal karyotyping, chromosomal microarray analysis (CMA), and trio whole-exome sequencing (WES). Sanger sequencing and quantitative fluorescence PCR (QF-PCR) were performed as affirmative experiments. In six families, a total of six pathogenic/likely pathogenic variations were identified in four genes including* SLC26A2, FGFR3, FLNB, *and* TMEM38B*. These variations caused disorders following autosomal dominant or autosomal recessive inheritance patterns, respectively. The results provided reliable evidence for the subsequent genetic counseling and reproductive options to these families. With its advantage in variation calling and interpreting, trio WES is a promising strategy for the investigation of fetal SDs in cases with normal karyotyping and CMA results. It has considerable prospects to be utilized in prenatal diagnosis.

## 1. Introduction

Skeletal dysplasias (SDs), a series of heterogeneous genetic disorders affecting approximately 2.3 to 4.5 of 10,000 births [1–3], are often hereditable and affect the growth, morphometry, and integrity of cartilage and/or bone. SDs are individually rare, but collectively they comprise a large group of disorders ranging from relatively mild anomalies to lethality. According to the 2015 Nosology and Classification of Genetic Skeletal Disorders [4], 436 genetic skeletal disorders were classified into 42 groups, associating with one or more of 364 genes. However, only a small part of these disorders has clear molecular pathogenesis [5]. Moreover, skeletal involvement may also occur in other multisystem syndromes [6]. Therefore, due to the clinical and genetic heterogeneity of SDs, it is very challenging to make a clear diagnosis, particularly in the prenatal diagnosis of fetal SDs.

Ultrasonography is still an indispensable first-line screening method. However, it has limitations, mainly in the differential diagnosis of similar SDs. In recent years, the cost of genetic testing techniques has been reduced, while their throughput has dramatically increased, which greatly benefits the precise diagnosis of fetal structural disorders [7–9]. Among all genetic testing methods, the trio WES strategy has the unique advantages in the efficiency of variation calling and the sensitivity of detecting* de novo* and compound heterozygous variants [9], particularly in cases with obvious structural abnormalities and normal karyotyping and CMA results.

In this study, in order to investigate the genetic cause of fetal SDs in eight pregnancies with nonconsanguineous parents, a sequential detection including trio WES was performed to make a clear diagnosis. Then* in silico* prediction on the functional impact of the identified novel variants was conducted.

## 2. Materials and Methods

### 2.1. Subjects

Research ethics board approval was obtained from the Human Ethics Committee of Chinese PLA General Hospital (approval no. S2018-066-01), and informed consent forms were signed by all recruited subjects. Between November 2016 and March 2018, we recruited eight families with pregnancies interrupted in their second or third trimesters due to fetal SDs based on clinical and sonographic diagnosis. Detailed information including maternal age, gestational weeks, and obstetric history was documented. Parental peripheral blood and fetal tissue or umbilical cord blood samples were obtained by routine methods during or after the procedure of odinopoeia.

### 2.2. Chromosome Karyotyping and CMA

All fetal specimens underwent conventional G-banded karyotyping test according to standard operation procedures to detect overall chromosomal anomalies. CMA tests with CytoScan 750K (Affymetrix Inc., USA) arrays were performed according to the manufacturer's manual workflow on all fetal specimens in order to investigate genomic copy number variants with clinical significance. Data was collected and analyzed by GeneChip® Scanner 3000 with AGCC software. The pathogenicity of detected variations was determined according to guidelines issued by the American College of Medical Genetics and Genomics (ACMG) in 2011 [10].

### 2.3. Whole-Exome Sequencing

Trio WES strategy was taken to identify the causal variants. 1*μ*g genomic DNA from 200*μ*l peripheral blood or 5-10 mg fetal tissue was extracted using a Qiagen DNA Blood Midi/Mini Kit (Qiagen GmbH, Hilden, Germany) according to manufacturer's protocol. DNA fragments were hybridized and captured by IDT's xGenExome Research Panel (Integrated DNA Technologies, San Diego, USA) according to manufacturer's protocol. The libraries were tested for enrichment by qPCR, and the size distribution and concentration were determined using an Agilent Bioanalyzer 2100 (Agilent Technologies, Santa Clara, CA, USA). The Novaseq6000 platform (Illumina, San Diego, USA), along with 150 bp pair-end reads, was used for the genomic sequencing of DNA. The sequencing reads were aligned to the human reference genome (hg19/GRCh37) using the Burrows-Wheeler Aligner tool and the PCR duplicates were removed by using Picard v1.57 (http://picard.sourceforge.net/). The Verita Trekker® Variants Detection System by Berry Genomics and the third-party software GATK (https://software.broadinstitute.org/gatk/) were employed for variant calling. Variant annotation and interpretation were conducted through the use of ANNOVAR [11] and the Enliven® Variants Annotation Interpretation System authorized by Berry Genomics. During trio analysis, potential monogenetic inheritance patterns, including* de novo*, autosomal recessive, autosomal dominant, X-linked recessive inheritance, mitochondrial, and, where possible, imprinted gene variations, were analyzed.

*In silico *analysis using Sorting Intolerant from Tolerant (SIFT) (http://sift.bii.a-star.edu.sg/) and Polymorphism Phenotyping V2 (http://genetics.bwh.harvard.edu/pph2/) was performed in order to calculate the pathogenicity index of all novel missense variants with unknown clinical significance. The variants were classified according to the ACMG guidelines for interpretation of genetic variants [12]. For pathogenic or likely pathogenic variations reported by trio WES, Sanger sequencing or quantitative fluorescence PCR (QF-PCR) was performed as a confirmatory experiment (See Supplementary Material 2 for detailed molecular data including primer sequences, reaction systems and amplification conditions). Homological analysis among species was performed using NCBI blast online software (https://blast.ncbi.nlm.nih.gov/Blast.cgi). Three-dimensional structure prediction was conducted through the use of Modeller V9.21 (https://salilab.org/modeller/).

## 3. Results

### 3.1. Clinical Features

In the eight families we recruited, the average age of gravidae was 31 (ranging from 24 to 38), and the average gestational week of these pregnancies was 20.9 (ranging from 16 to 29). None of the couples was consanguineous, and all couples claimed to have no family history of genetic disorders. Major clinical manifestations and information of these pregnancies were listed in Table 1 (See detailed clinical data of all eight families in Supplementary Material 1).

### 3.2. Genetic Analysis

**Figure 1:**
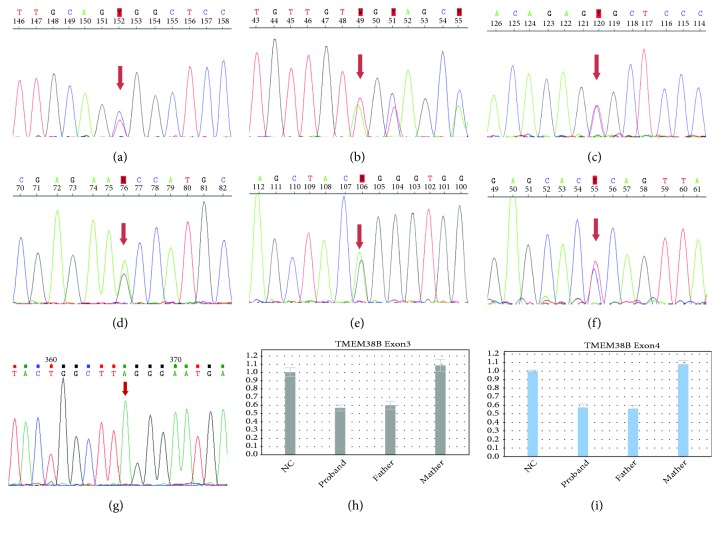
Results of Sanger sequencing and QF-PCR: (a) a single-base substitution in* SLC26A2 *(c.292T>C); (b) a three-base deletion in* SLC26A2 *(c.1018_1020del); (c) a single-base substitution in* FGFR3 *(c.742C>T); (d) a single-base substitution in* FLNB *(c.601G>A); (e) a single-base substitution in* FGFR3 *(c.1138G>A); (f) a single-base substitution in* FLNB *(c.685T>C); (g) a single-base substitution in exon 3 of* TMEM38B *(c.344C>A); (h) one copy loss of exon 3 in* TMEM38B*; (i) one copy loss of exon 4 in* TMEM38B.*

Results of karyotyping and CMA for all fetal specimens from eight pregnancies were normal. Variations with clinical significance detected by trio WES were listed in Table 2, and results of corresponding Sanger sequencing and QF-PCR were demonstrated in Figure 1.

In Family 1, a compound heterozygous variation in* SLC26A2* comprising c.292T>C (Figure 1(a)) and c.1018_1020del (Figure 1(b)) was identified. The two variants were inherited from the mother (c.292T>C) and father (c.1018_1020del) of the proband fetus. Sanger sequencing revealed that the two normal daughters were one carrier of c.1018_1020del as the father and the other one as wild type.

Four* de novo* variations were identified including* FGFR3*:c.742C>T (Figure 1(c)) in Family 2,* FLNB*: c.601G>A (Figure 1(d)) in Family 3,* FGFR3*: c.1138G>A (Figure 1(e)) in Family 5, and* FLNB*: c.685T>C (Figure 1(f)) in Family 6.

In Family 8, the compound heterozygous variations detected in* TMEM38B*, c.344C>A (Figure 1(g)), and loss 1 (exon: 3-4) (Figures 1(h) and 1(i) and Supplementary Figure 2-1), were inherited from the mother and father, respectively. The normal daughter was a carrier of c.344C>A, like her mother.

Four novel variations were identified in this study, namely,* FLNB*: c.601G>A,* SLC26A2*: c.292T>C,* TMEM38B*: c.344C>A, and* TMEM38B*: loss 1 (exon: 3-4).* In silico* pathogenicity prediction was conducted on two novel missense variants (*FLNB*: c.601G>A and* SLC26A2*: c.292T>C), and the results from SIFT and PolyPhen V2 indicated them as “deleterious/probably damaging” (Table 2).

**Figure 2:**
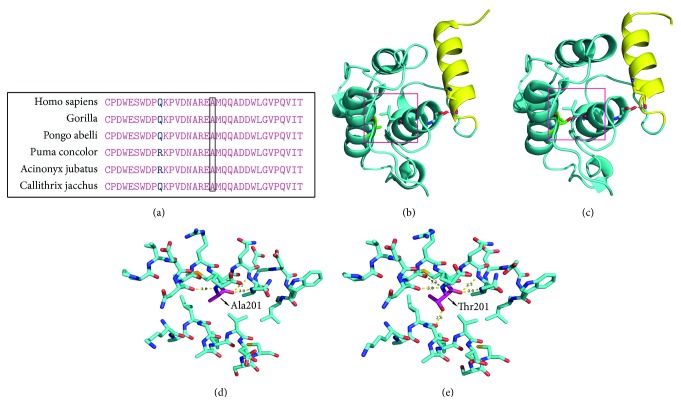
Biophysical analyses of the* FLNB*: c.601G>A(p.Ala201Thr) variation: (a) the conservation of* FLNB* Ala201 between multiple species; (b) part of the three-dimensional structure of the wild type* FLNB*, with the CH2 subdomain in blue and the actin-binding site 3 in yellow; (c) part of the three-dimensional structure of the mutant* FLNB*, with the CH2 subdomain in blue and the actin-binding site 3 in yellow; (d) the detailed red block in (b) showing Ala201; (e) the detailed red block in (c) showing Thr201 and the extra hydrogen bonds among T201 and L181, K152, and S177.

The results of NCBI blast showed that* FLNB*-Ala201 and* SLC26A2*-Trp98 amino acids were highly conserved among species (Figure 2(a), also see Supplementary Figure 2-2). Additionally, the three-dimensional structure-prediction result of the mutant protein showed that* FLNB*: c.601G>A (p.Ala201Thr) may cause the formation of two extra hydrogen bonds (T201-L181 and K152-S177; see Figure 2(e)).

## 4. Discussion

The clinical heterogeneity of skeletal dysplasias is strong. Some fetal phenotypes are relatively vague and some fetal phenotypes may not have obvious manifestations until the third trimester, which leads to the challenge in ultrasonic and differential diagnosis. Meticulously designed strategy of genetic testing may help solve this problem. Several studies have discussed the advantage of trio WES with respect to the efficiency of variation screening [9, 13, 14], which makes possible the application to prenatal diagnosis.

The* SLC26A2 *(MIM *∗*606718) protein transports ions, particularly sulfate ions, across cell membranes that help cartilage to produce proteoglycans [15]. The impaired function of the* SLC26A2* product would be expected to lead to undersulfation of proteoglycans in the cartilage matrix and thereby cause a spectrum of SDs, including achondrogenesis IB (ACG-IB,MIM #600972), atelosteogenesis II (MIM #256050), De la Chapelle dysplasia (MIM #256050), diastrophic dysplasia (DTD, MIM # 222600) and epiphyseal dysplasia, multiple, i.e., 4 (MIM # 226900). Superti-Furga et al. first established an association between* SLC26A2* and ACG-IB [16]. Among the compound heterozygous mutations detected in Family 1, the variant* SLC26A2*: c.1018_1020del (p.Val340del) was known as pathogenic, and its homozygous mutation was first reported to cause ACG-IB by Superti-Furga et al. [17]. Contrastingly, the variant* SLC26A2*: c.292T>C (p.Trp98Arg) (no. Rs753193118 in the dbSNP database) has not yet been reported as pathogenic. Its frequency in the gnomAD database is 4.06×10^−6^ (http://gnomad.broadinstitute.org/), and it was predicted as deleterious by the SIFT and PolyPhen software programs. We then interpreted c.292T>C as likely pathogenic according to the ACMG criteria with evidence levels PM2+PM3+PP2+PP3+PP4. The relationship between different types of homozygous or complex heterozygous mutations and the severity of corresponding disease phenotypes was discussed [17]. It is believed that DTD is associated with reduced* SLC26A2* expression, while ACG-IB results from the null mutations of it. Thus, the ability to predict the specific disease type of the fetus in Family 1 depends on the revelation of the expression level of* SLC26A2*.

*FGFR3 *(MIM *∗*134934) belongs to the fibroblast growth factor family which plays an important role in cell proliferation and differentiation, angiogenesis, wound healing, and embryo development (https://ghr.nlm.nih.gov/gene/FGFR3). It is believed that the* FGFR3* protein regulates bone growth by limiting ossification progress, particularly in long bones [18]. The two variants detected in Family 2 (*FGFR3*: c.742C>T) and Family 5 (*FGFR3*: c.1138G>A) have been reported many times as pathogenic [19–22]. However, these two variants lead to different disorders (Table 2) that have different phenotypes and prognoses, which are difficult to distinguish from fetal sonographic indications.* FGFR3*: c.742C>T is one of the two most common mutations in thanatophoric dysplasia, type I (MIM #187600), while* FGFR3*: c.1138G>A contributes to more than 90% of the condition in achondroplasia (MIM #100800) patients [23, 24]. These results indicate that genetic analysis is of great significance for prognosis prediction and clinical consultation to these families with variations in identical genes.

Filamins, including* FLNB *(MIM *∗*603381), are actin-binding proteins that also interact with multiple receptors and intracellular proteins, which in turn regulate cytoskeleton-dependent cell proliferation, differentiation, and migration [25]. Previous studies have shown that heterozygous missense variations in* FLNB *lead to a spectrum of severe SDs including atelosteogenesis type I (AOI, MIM #108720), atelosteogenesis type III (AOIII, MIM #108721), Boomerang dysplasia (MIM #112310), Larsen syndrome (MIM #150250), and spondylocarpotarsal synostosis syndrome (MIM #272460). In our study, the variant in Family 3,* FLNB*: c.601G>A (p.Ala201Thr), has not been previously reported, but it shares the same amino acid that is affected by a variant,* FLNB*: c.602C>T (p.Ala201Val), as detected in a neonate with AOIII [26]. We then interpreted c.601G>A as likely pathogenic according to the ACMG criteria (PS2+PM2+PM5+PP3). Sawyer et al. pointed out that missense mutations in particular regions of* FLNB* may follow the mechanism of gain of function and enhance its binding affinity with actin [27]. The structure-prediction result in our study is likely consistent with this concept. The identified variation* FLNB*: c.685T>C (p.Ser229Pro) in Family 6 was previously reported as a pathogenic variant responsible for Larsen syndrome [28]. These two variations in our study are located within the CH2 subdomain of the actin-binding domain (ABD) in* FLNB*, and they may cause the dysregulation of actin-filamin interaction, which associates to the skeletal phenotype spectrum of the probands.

*TMEM38B *(MIM *∗*611236) encodes trimeric intracellular cation-B (TRIC-B) protein, which expresses differently in various tissues and cells of animals. TRIC-B channels act as counter-ion channels that function in synchronization with* Ca*^2+^ release from intracellular stores [29]. Pathogenic variations in* TMEM38B *were reported to cause a rare autosomal recessive type of osteogenesis imperfecta (OIXIV, MIM #615066). Patients of OIXIV usually develop moderately severe OI. They have various fracture frequencies, mildly to moderately short stature, and gray-to-blue sclera but no occurrence of dental defects [30]. To our knowledge, six different mutations of TMEM38B have been reported in previous studies [30, 31]. The compound heterozygous variation detected in Family 8 consists of two novel variants: one (c.344C>A) can cause premature termination of protein translation, and the other (loss 1 (exon: 3-4)) may result in truncated protein. Each of those variants has a serious impact on the function of TRIC-B protein and is classified as pathogenic according to ACMG criteria. Nevertheless, the mechanism of variable expressivity in different OIXIV cases remains to be studied. Moreover, the detection of these two variants expanded the mutant spectrum of OIXIV and will be very helpful in the continued investigation of* TMEM38B* function.

In the remaining two families (4 and 7), no variation with clear clinical significance was detected. Thus, further research is essential. Pathogenic variations may be identified after reanalysis over an extensive period of time, particularly given the emergence of new disease-causing genes, and new mechanisms or pathogenesis may be discovered through in-depth investigation.

This study helped SD families to identify the cause and accurately assessed the risks inherent with further pregnancies. Each participating family with positive results had a different inheritance pattern of disease and therefore a different risk of recurrence during pregnancy. The families corresponding to autosomal recessive pattern (Families 1 and 8) have a 25% risk in each pregnancy. The families corresponding to autosomal dominant pattern (Families 2, 3, 5, and 6) commonly have minimal risk of recurrence, but it will be relatively higher if there is germinal mosaicism [32, 33]. This issue should be considered in the clinical consultation and subsequent pregnancy examination.

## 5. Conclusions

Specific laboratory diagnosis is difficult with respect to cases that involve skeletal dysplasias, given the low incidence of SDs as well as their strong clinical and genetic heterogeneity, particularly in the field of prenatal diagnosis. Comprehensive application of multiple genetic techniques can effectively improve the diagnosis rate of SDs. Thus, the trio WES strategy provides a robust methodological supplement in case there is lack of clear imageological evidence and sufficient clinical experience.

## Figures and Tables

**Table 1:** Clinical information of recruited SD pregnancies.

Subject	Maternal age (years)	Gestational weeks	Clinical information^*※*^	Fetal sample origin
Family 1	31	16	G_5_P_2_A_1_: 2 daughters with normal phenotype; 2 singleton pregnancies with limb shortening	Umbilical cord tissue

Family 2	31	22	G_1_P_0_: limb shortening	Umbilical cord blood

Family 3	29	16	G_3_P_0_A_2_: 2 miscarriages; 1 SD pregnancy diagnosed at 13 gestational weeks after IVF-ET	Umbilical cord tissue

Family 4	31	22	G_2_P_0_: 1 induced abortion; 1 pregnancy with thick NT(0.30cm at 12 weeks), micrognathia, ulnar and osteogenic dysplasia, abnormal hand shape	Umbilical cord blood

Family 5	38	16	G_2_P_1_: 1 daughter with normal phenotype; 1 pregnancy with limb shortening	Umbilical cord tissue

Family 6	28	23	G_1_P_0_: osteogenic dysplasia at 22 gestational weeks	Umbilical cord tissue

Family 7	24	23	G_3_P_0_A_1_: 1 miscarriage; 2 pregnancies with thick NT and femur shortening	Umbilical cord tissue

Family 8	36	29	G_3_P_1_; 1 daughter with normal phenotype; 1 pregnancy with osteogenic dysplasia and angled left femur at 23 gestational weeks	Umbilical cord tissue

^*※*^G: gravida; P: para; A: abortus; IVF-ET: In Vitro Fertilization-Embryo Transfer.

**Table 2:** Information of detected variations.

Subject	Gene	Variation	Disorder	Inheritance pattern^*※*^	Variation effect (origin)	Global MAF^*※*^ (dbSNP)	SIFT score	PolyPhen2 score	Variation attribute (ACMG evidence levels)
Family 1	*SLC26A2*	c.292T>C (p.Trp98Arg)	Achondrogenesis IB or diastrophic dysplasia	AR	Missense(mother)	C=0.0000/0(TWINSUK); C=0.0000/1(GnomAD_exomes); C=0.0000/1(TOPMED); C=0.0000/1(ExAC); C=0.0003/1(ALSPAC)	0	1	Likely pathogenic (PM2 + PM3 + PP2 + PP3 + PP4)
		c.1018_1020del (p.Val340del)			In-frame deletion(father)	/	/	/	Pathogenic (PS1 + PM2 + PM4 + PP4 + PP5)
Family 2	*FGFR3*	c.742C>T (p.Arg248Cys)	Thanatophoric dysplasia, type I	AD	Missense (de novo)	/	/	/	Pathogenic (PS1 + PS2 + PM1 + PM2)
Family 3	*FLNB*	c.601G>A (p.Ala201Thr)	Atelosteogenesis, type I or III	AD	Missense (de novo)	/	0	0.996	Likely pathogenic (PS2 + PM2 + PM5 + PP3)
Family 5	*FGFR3*	c.1138G>A (p.Gly380Arg)	Achondroplasia	AD	Missense variant (de novo)	A=0.0000/1(TOPMED)	/	/	Pathogenic (PS1 + PS2 + PM1 + PM2)
Family 6	*FLNB*	c.685T>C (p.Ser229Pro)	Larsen syndrome	AD	Missense (de novo)	/	/	/	Pathogenic (PS1 + PS2 + PM2)
Family 8	*TMEM38B*	c.344C>A (p.S115X)	Osteogenesis imperfecta, type XIV	AR	Stop gained(Mother)	/	/	/	Pathogenic (PVS1 + PM2 + PP4)
		loss 1 (exon:3-4)			Exon loss (Father)	/	/	/	Pathogenic (PVS1 + PM2 + PM4 + PP4)

^*※*^AD: autosomal dominant; AR: autosomal recessive; MAF: minor allele frequency.

## Data Availability

The authors provided a comprehensive molecular data in Supplementary Material 2. If necessary, the authors are willing to upload the raw data such as Sanger sequence files according to the editor's discretion.

## Supplementary Materials

Supplementary Materials The supplementary materials, in two parts, are provided along with the manuscript in the form of a ZIP file, which included detailed clinical data and additional molecular data. The contents of the supplementary materials are referenced at appropriate points within the manuscript.

## References

[1] Dighe M., Fligner C., Cheng E., Warren B., Dubinsky T. (2008). Fetal skeletal dysplasia: an approach to diagnosis with illustrative cases. *RadioGraphics*.

[2] Lachman R. S., Rappaport V. (1990). Fetal imaging in the skeletal dysplasias. *Clinics in Perinatology*.

[3] Orioli I. M., Castilla E. E., Barbosa-Neto J. G. (1986). The birth prevalence rates for the skeletal dysplasias. *Journal of Medical Genetics*.

[4] Bonafe L., Cormier-Daire V., Hall C. (2015). Nosology and classification of genetic skeletal disorders: 2015 revision. *American Journal of Medical Genetics Part A*.

[5] Geister K. A., Camper S. A. (2015). Advances in skeletal dysplasia genetics. *Annual Review of Genomics and Human Genetics*.

[6] Offiah A. C. (2015). Skeletal dysplasias: an overview. *Endocrine Development*.

[7] Barkova E., Mohan U., Chitayat D. (2015). Fetal skeletal dysplasias in a tertiary care center: Radiology, pathology, and molecular analysis of 112 cases. *Clinical Genetics*.

[8] Zhang W., Taylor S. P., Ennis H. A. (2018). Expanding the genetic architecture and phenotypic spectrum in the skeletal ciliopathies. *Human Mutation*.

[9] Fu F., Li R., Li Y. (2018). Whole exome sequencing as a diagnostic adjunct to clinical testing in fetuses with structural abnormalities. *Ultrasound in Obstetrics & Gynecology*.

[10] Kearney H. M., Thorland E. C., Brown K. K., Quintero-Rivera F., South S. T. (2011). American College of Medical Genetics standards and guidelines for interpretation and reporting of postnatal constitutional copy number variants. *Genetics in Medicine*.

[11] Wang K., Li M., Hakonarson H. (2010). ANNOVAR: functional annotation of genetic variants from high-throughput sequencing data. *Nucleic Acids Research*.

[12] Richards S., Aziz N., Bale S. (2015). Standards and guidelines for the interpretation of sequence variants: a joint consensus recommendation of the American college of medical genetics and genomics and the association for molecular pathology. *Genetics in Medicine*.

[13] Hegde M., Santani A., Mao R., Ferreira-Gonzalez A., Weck K. E., Voelkerding K. V. (2017). Development and validation of clinical whole-exome and whole-genome sequencing for detection of germline variants in inherited disease. *Archives of Pathology & Laboratory Medicine*.

[14] Al-Mubarak B., Abouelhoda M., Omar A. (2017). Whole exome sequencing reveals inherited and de novo variants in autism spectrum disorder: a trio study from Saudi families. *Scientific Reports*.

[15] Alper S. L., Sharma A. K. (2013). The SLC26 gene family of anion transporters and channels. *Molecular Aspects of Medicine*.

[16] Superti-Furga A., Hastbacka J., Cohn D. H. (1995). Defective sulfation of proteoglycans in achondrogenesis type 1B is caused by mutations in the DTDST gene: the disorder is allelic to diastrophic dysplasia. *American Journal of Human Genetics*.

[17] Superti-Furga A., Hastbacka J., Wilcox W. R. (1996). Achondrogenesis type IB is caused by mutations in the diastrophic dysplasia sulphate transporter gene. *Nature Genetics*.

[18] Matsushita T., Wilcox W. R., Chan Y. Y. (2009). FGFR3 promotes synchondrosis closure and fusion of ossification centers through the MAPK pathway. *Human Molecular Genetics*.

[19] Tavormina P. L., Shiang R., Thompson L. M. (1995). Thanatophoric dysplasia (types I and II) caused by distinct mutations in fibroblast growth factor receptor 3. *Nature Genetics*.

[20] Wilcox W. R., Tavormina P. L., Krakow D. (1998). Molecular, radiologic, and histopathologic correlations in thanatophoric dysplasia. *American Journal of Medical Genetics*.

[21] Saito H., Sekizawa A., Morimoto T., Suzuki M., Yanaihara T. (2000). Prenatal DNA diagnosis of a single-gene disorder from maternal plasma. *The Lancet*.

[22] Wyrobek A. J., Eskenazi B., Young S. (2006). Advancing age has differential effects on DNA damage, chromatin integrity, gene mutations, and aneuploidies in sperm. *Proceedings of the National Acadamy of Sciences of the United States of America*.

[23] Bellus G. A., Hefferon T. W., de Ortiz Luna R. I. (1995). Achondroplasia is defined by recurrent G380R mutations of FGFR3. *American Journal of Human Genetics*.

[24] Rousseau F., Bonaventure J., Legeai-Mallet L. (1994). Mutations in the gene encoding fibroblast growth factor receptor-3 in achondroplasia. *Nature*.

[25] Hu J., Lu J., Lian G., Ferland R. J., Dettenhofer M., Sheen V. L. (2014). Formin 1 and filamin B physically interact to coordinate chondrocyte proliferation and differentiation in the growth plate. *Human Molecular Genetics*.

[26] Farrington-Rock C., Firestein M. H., Bicknell L. S. (2006). Mutations in two regions of FLNB result in atelosteogenesis I and III. *Human Mutation*.

[27] Sawyer G. M., Clark A. R., Robertson S. P., Sutherland-Smith A. J. (2009). Disease-associated substitutions in the filamin B actin binding domain confer enhanced actin binding affinity in the absence of major structural disturbance: Insights from the crystal structures of filamin B actin binding domains. *Journal of Molecular Biology*.

[28] Daniel P. B., Morgan T., Alanay Y. (2012). Disease-associated mutations in the actin-binding domain of filamin B cause cytoplasmic focal accumulations correlating with disease severity. *Human Mutation*.

[29] Yazawa M., Ferrante C., Feng J. (2007). TRIC channels are essential for Ca2^+^ handling in intracellular stores. *Nature*.

[30] Ichimura A., Takeshima H. (2016). TRIC-B mutations causing osteogenesis imperfecta. *Biological & Pharmaceutical Bulletin*.

[31] Caparros-Martin J. A., Aglan M. S., Temtamy S. (2017). Molecular spectrum and differential diagnosis in patients referred with sporadic or autosomal recessive osteogenesis imperfecta. *Molecular Genetics & Genomic Medicine*.

[32] Van der Meulen M. A., Van der Meulen M. J. P., Te Meerman G. J. (1995). Recurrence risk for germinal mosaics revisited. *Journal of Medical Genetics*.

[33] Natacci F., Baffico M., Cavallari U. (2008). Germline mosaicism in achondroplasia detected in sperm DNA of the father of three affected sibs. *American Journal of Medical Genetics Part A*.

