# Supplementary material for: Genetic Analysis in Fetal Skeletal Dysplasias by Trio Whole-Exome Sequencing
Source: Biomed Res Int. 2019 May 14;2019:2492590. doi: 10.1155/2019/2492590 (PMC6537022; doi:10.1155/2019/2492590)

**Supplementary Material 1**

**Clinical data**

**Family1**: The pregnant woman was 30 years old, and her husband was 31. They had a daughter with normal mental and physical phenotypes in 2009. After that, they had a conception of a fetus with thick nuchal translucency(NT) and shortened limbs in 2013, and induced abortion was conducted at 14 weeks of gestation. Then in 2016, they had another affected fetus as the previous one according to ultrasonic diagnosis, and referred to our center for genetic diagnosis at 15^+5^ weeks of gestation. Then induced abortion was carried out at 16 weeks of gestation, and the fetus' sample was collected. Results of prenatal ultrasonography were demonstrated below (Supplementary Fig. 1-1).

Afterwards, they had a miscarriage at 12 weeks of gestation in 2017. Then, they gave birth to another normal daughter in 2018. The pedigree information is shown below (Supplementary Fig. 1-1).

Specimens from the proband, the parents, and the two normal daughters were collected and subjected to genetic analysis.

**Supplementary Fig. 1-1** Prenatal ultrasonography of the proband fetus (above). Pedigree information of Family 1 (below).


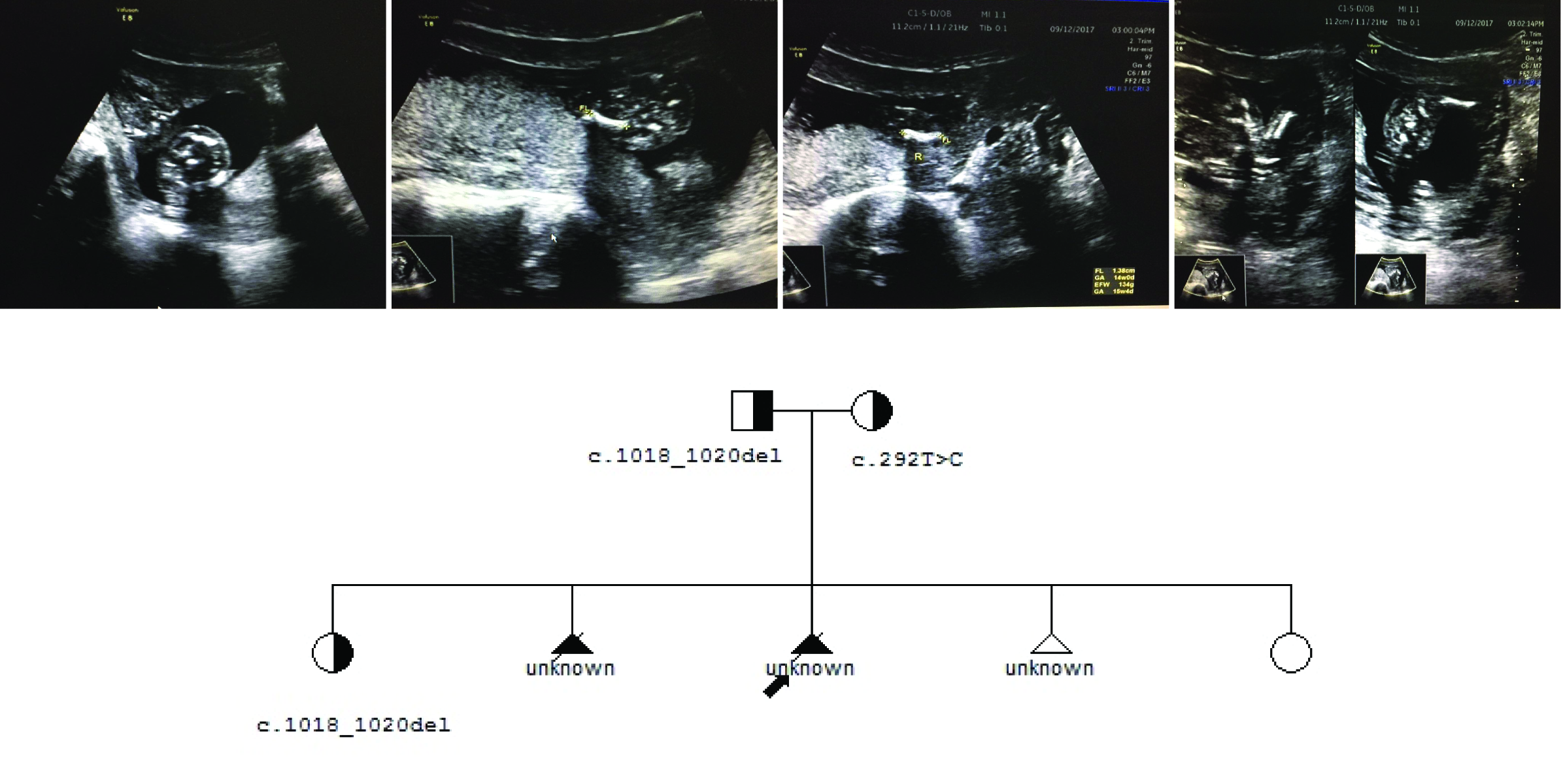


**Family 2:** The pregnant woman was 30 years old, and her husband was 32.This was their first pregnancy. The fetus was diagnosed with shortened and slightly curved limbs and polyhydramnios by ultrasonography at 22 weeks of gestation. During the prenatal diagnosis procedure, we collected trio samples (umbilical cord blood and parental peripheral blood)for further tests. The couple decided to terminate this pregnancy at 23 weeks of gestation. One year later when we followed up with this couple, this women developed renal cancer and had her right kidney removed.

The ultrasonic image and pedigree information were shown below (Supplementary Fig. 1-2).

**Supplementary Fig. 1-2** Ultrasonography results of the proband fetus (above) and pedigree information of Family 2 (below).


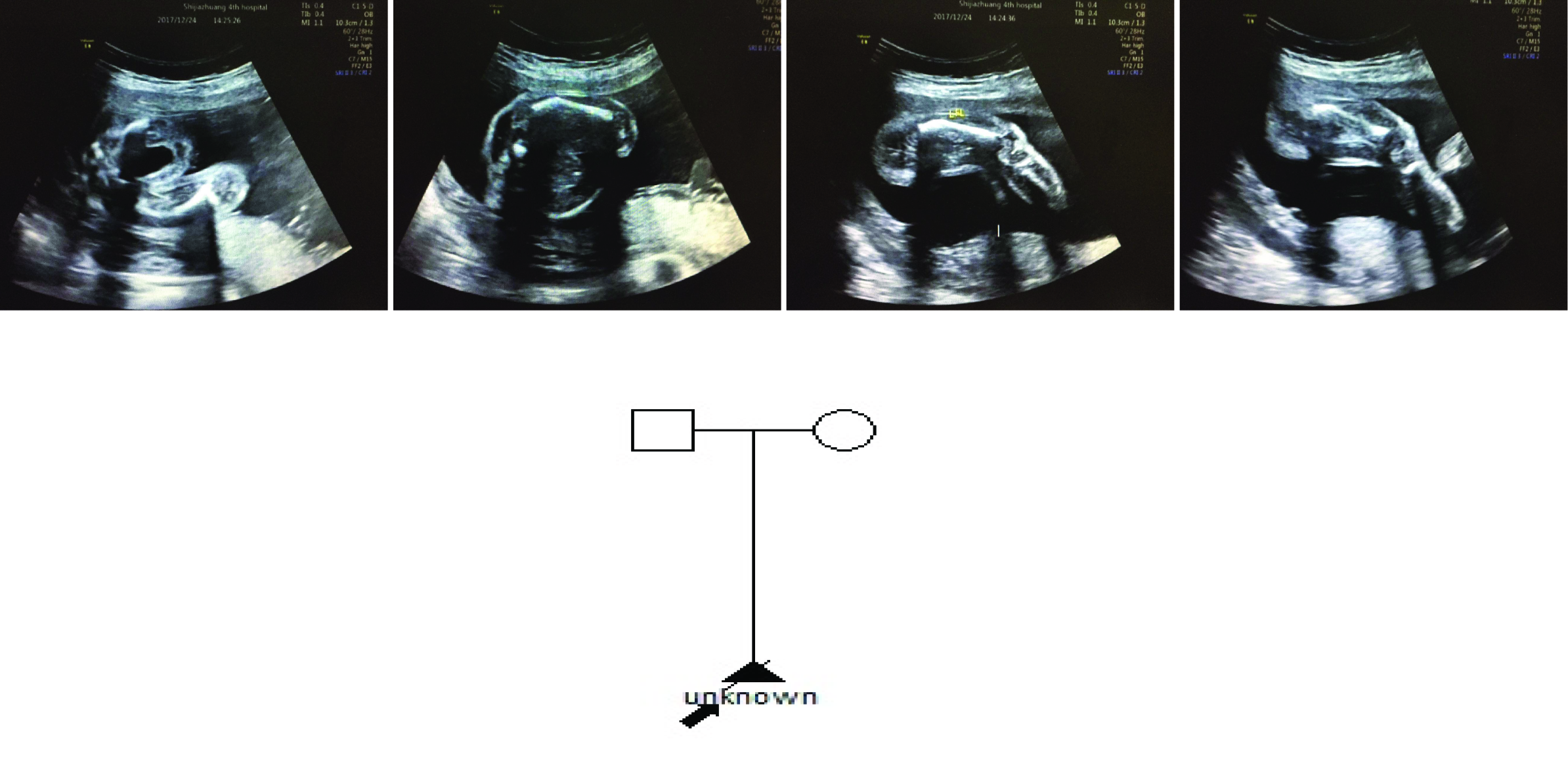


**Family 3:** The pregnant woman was 29 years old, and her husband was 36. They had two miscarriages before this pregnancy. This time, the conception was via in vitro fertilization and embryo transfer techniques. The fetus was diagnosed with seemingly skeletal dysplasia at 13 weeks of gestation. After abortion, we collected trio samples for this study.

The ultrasonic image and pedigree information were shown below (Supplementary Fig. 1-3).

**Supplementary Fig. 1-3** Ultrasonography results of the proband fetus (4 images above) and pedigree information of Family 3 (below).


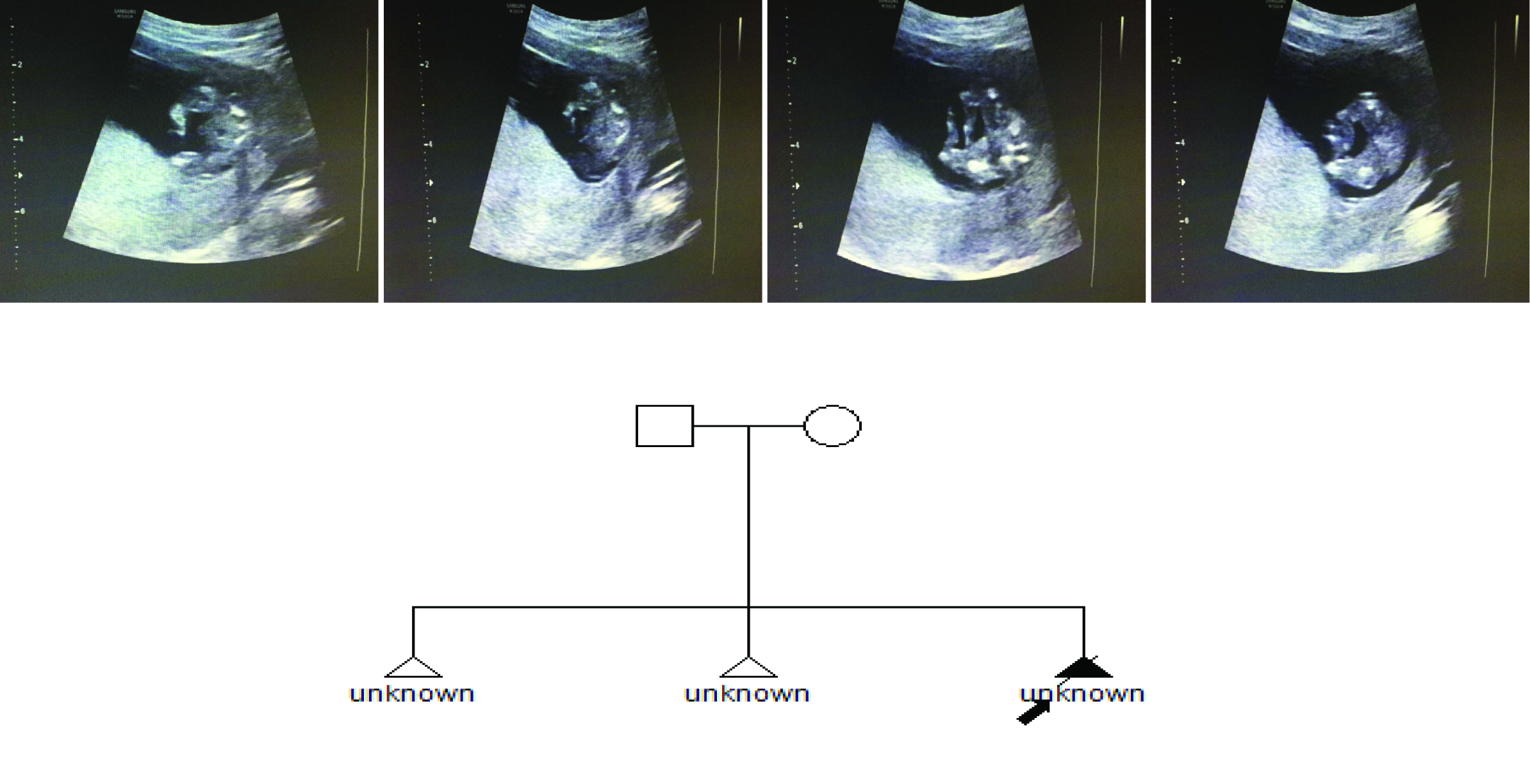


**Family 4:** The pregnant woman was 31 years old, and her husband was 33. In 2013, they had anectopic pregnancy, and the right fallopian tube of this woman was removed along with induced abortion. The fetus was diagnosed with thick NT at 12 weeks of gestation, abnormal hand shape at 16 weeks; and micrognathia, ulnar and osteogenic dysplasia, and abnormal hand shape at 22 weeks. During the prenatal diagnosis procedure, we collected trio samples (umbilical cord blood and parental peripheral blood)for this study.

The pedigree information was shown below (Supplementary Fig. 1-4).

**Supplementary Fig. 1-4** Pedigree information of Family 4.


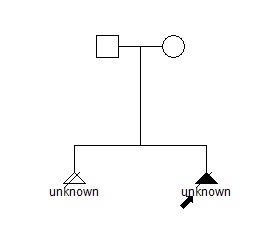


**Family 5:**The pregnant woman was 38 years old, and her husband was 39. They had a normal daughter in 2009. In this pregnancy, the fetus was diagnosed with shortened limbs at 15 weeks of gestation. After abortion, we collected trio samples for this study.

The pedigree information was demonstrated below (Supplementary Fig. 1-5).

**Supplementary Fig. 1-5** Pedigree information of Family 5.


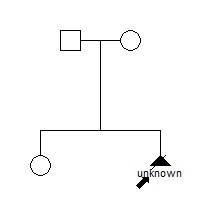


**Family 6:** The pregnant woman and her husband were both 28 years old. This was their first pregnancy. The fetus was diagnosed with osteogenic dysplasia and seemingly occult spina bifida at 22 weeks of gestation via ultrasonography. Induced abortion was conducted at 23 weeks of gestation. Afterwards, we collected trio samples for this study.

The aborted fetus and pedigree information was demonstrated below (Supplementary Fig. 1-6).

**Supplementary Fig. 1-6** Images of the aborted proband fetus (3 images above) and pedigree information of Family 6 (below).


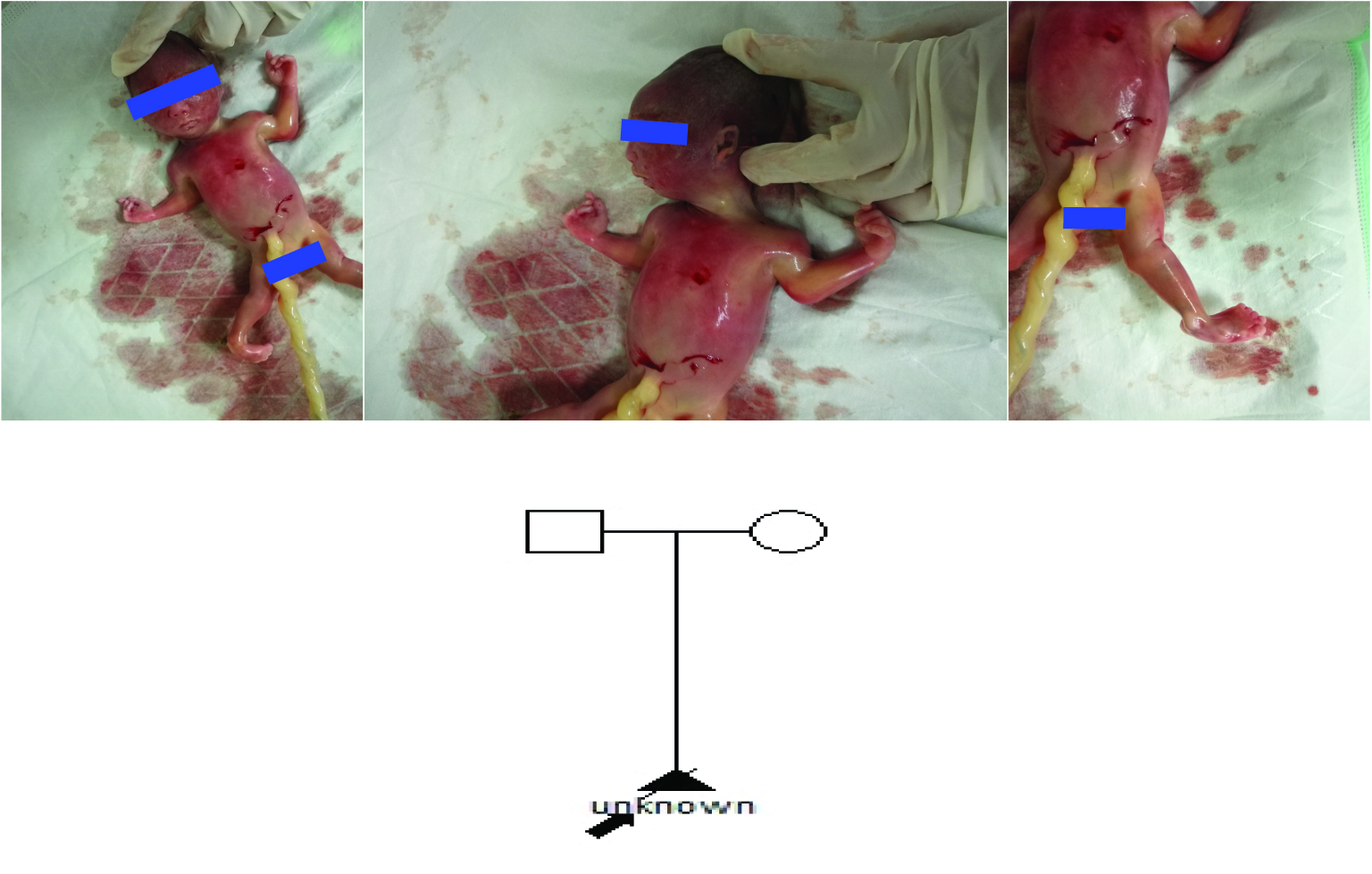


**Family 7:** The pregnant woman was 24 years old, and her husband was 26. They had a miscarriage in 2013. In 2015, they had an induced abortion because the fetus was diagnosed with cystic hygroma and short femur. In this pregnancy, the fetus was diagnosed with thick NT at 12 weeks of gestation, and short femur and stiffened leg joints at 22 weeks of gestation. Trio specimens were collected after the abortion procedure.

The ultrasonic image and pedigree information were shown below (Supplementary Fig. 1-7).

**Supplementary Fig. 1-7** Ultrasonography results of the proband fetus (3 images above) and pedigree information of Family 7 (below).


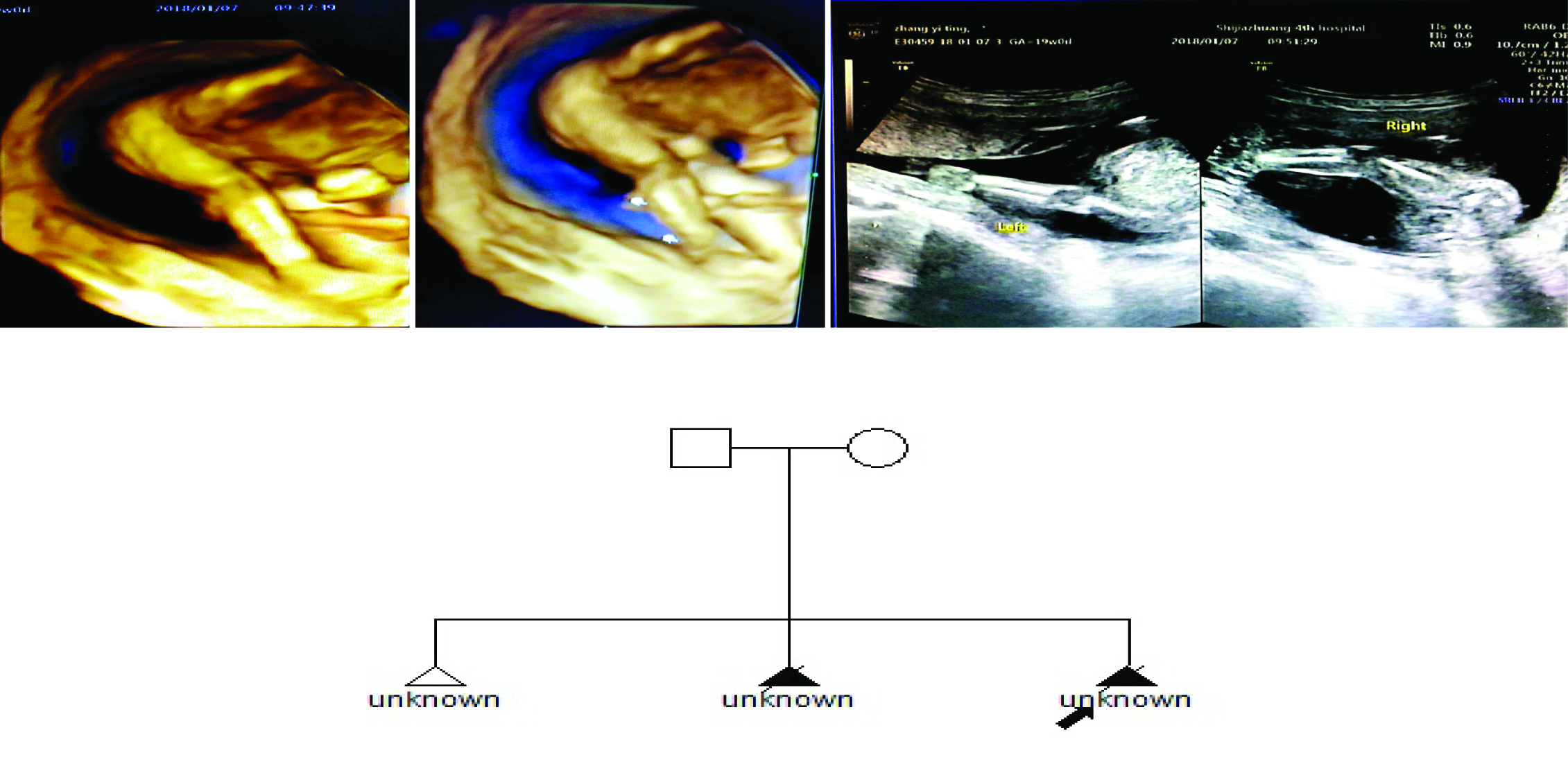


**Family 8:** The pregnant woman was 36 years old, and her husband was 44. They had an induced abortion in early trimester in 2004. Afterwards, they had a normal daughter born in 2006. In this pregnancy, the fetus was diagnosed with osteogenic dysplasia and angled left femur at 22^+^ weeks of gestation, and was aborted at 29 weeks. Trio specimens were collected after the abortion procedure.

The ultrasonic images, images of the aborted fetus and pedigree information were demonstrated below (**Supplementary Fig. 1-8**).

**Supplementary Fig. 1-8** Ultrasonography results and images (left 3 images above) after induced abortion of the proband fetus (far right image above), and the pedigree information of Family 8 (below).


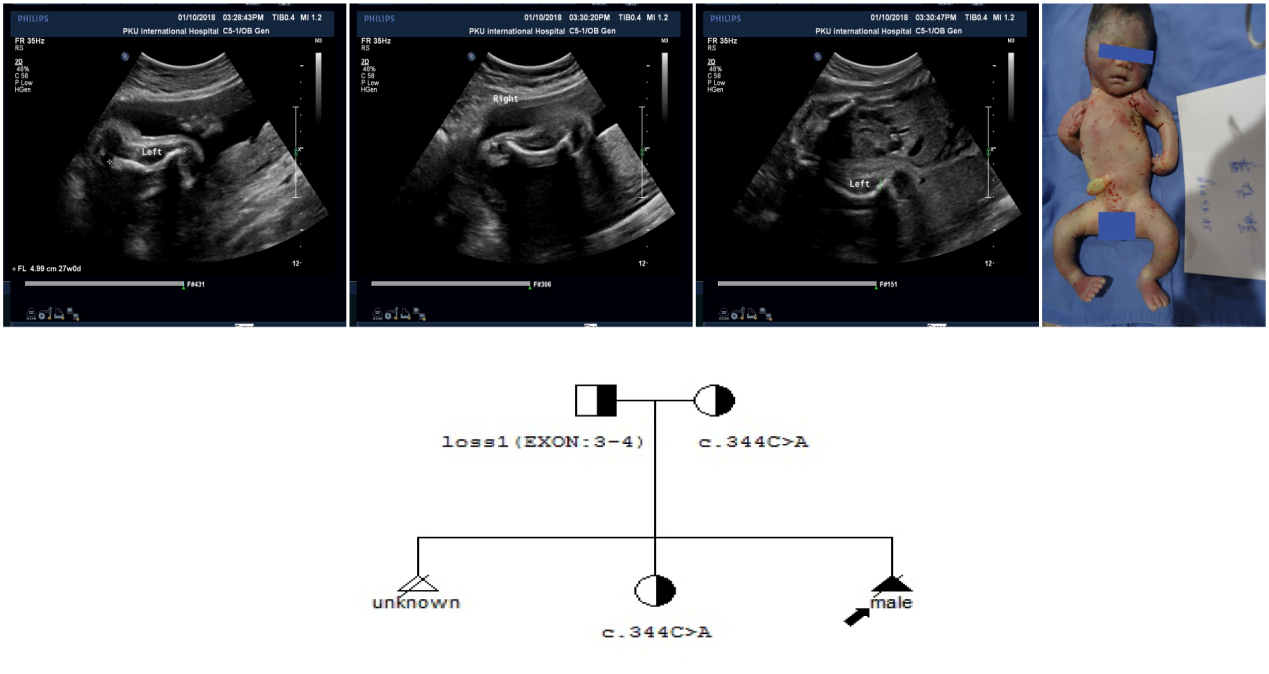

Supplement: Supplementary Materials — The supplementary materials, in two parts, are provided along with the manuscript in the form of a ZIP file, which included detailed clinical data and additional molecular data. The contents of the supplementary materials are referenced at appropriate points within the manuscript. [file 2492590.f1.zip › 2492590.f1/Supplementary Material 1-clinical data.docx]
