# Supplementary material for: Genetic Analysis in Fetal Skeletal Dysplasias by Trio Whole-Exome Sequencing
Source: Biomed Res Int. 2019 May 14;2019:2492590. doi: 10.1155/2019/2492590 (PMC6537022; doi:10.1155/2019/2492590)
Supplement: Supplementary Materials — The supplementary materials, in two parts, are provided along with the manuscript in the form of a ZIP file, which included detailed clinical data and additional molecular data. The contents of the supplementary materials are referenced at appropriate points within the manuscript. [file 2492590.f1.zip › 2492590.f1/Supplementary Material 2-molecular data.doc]

**Supplementary Material 2**

**Molecular Data**

**A.** In the following table (Supplementary Table 2-1), all primers used in Sanger sequencing and QF-PCR were listed. In addition, in the right two columns, we listed corresponding reaction system No. and amplification condition No. which were stated in the tables attached below (**Supplementary Table 2-2~8**).

**Supplementary Table 2-1**. Primers used in Sanger sequencing and QF-PCR.

| Family No | Gene | Target Variation | Primer sequences | Reaction system No. | Amplification condition No. |
| --- | --- | --- | --- | --- | --- |
| 1 | *SLC26A2* | c.292T>C | Forward: GGGATCCATCTGGAACTTCA  Reverse: ACGGGAGGTACCCAAGAGAA | 1 | 1 |
| 1 | *SLC26A2* | c.1018_1020del | Forward: TGTGATCTTATCACCAGCCTTT  Reverse: CAGCCACACTAGGAATTAGGTTC | 1 | 1 |
| 2 | *FGFR3* | c.742C>T | Forward: GGAGCCTGGTCATGGAAAG  Reverse: AGCACGGTAACGTAGGGTGT | 2 | 2 |
| 3 | *FLNB* | c.601G>A | Forward: GGGGTTAGTTTAGGCACATGG  Reverse: AAGGCATCCACAGAACAAGC | 1 | 1 |
| 5 | *FGFR3* | c.1138G>A | Forward: GCGTTACTGACTGCGAGACC  Reverse: AGGCAGCTCAGAACCTGGTA | 2 | 2 |
| 6 | *FLNB* | c.685T>C | Forward: GGCATTTGCAAACACTTCAA  Reverse: AACACCAGAGGACCAGCACT | 1 | 1 |
| 8 | *TMEM38B* | c.344C>A | Forward: ACTGGATTCAGCACTTAGCTTCT  Reverse: CACTCAGGCTCAAAATAATGGCT | 3 | 3 |
| 8 | *TMEM38B* | Loss1 (Exon3) | Forward: AATGAAGGAAGTGACCAGAACTTG  Reverse: GTCAATATTACCTCGGGCCCATC | 4 | 4 |
| 8 | *TMEM38B* | Loss1 (Exon4) | Forward: AGAAGGTGATGAATGGCTGAAGA  Reverse: TATTCCACCCACATTTAAAGTCCC | 4 | 4 |

**Supplementary Table 2-2**. Reaction system 1 (for one reaction)

| Reagent | Volume (µL) |
| --- | --- |
| ddH_2_O | 9.5 |
| reaction mixture | 12.5 |
| 5µM Primers | 1 forward and 1 reverse |
| template (>10ng/ul) | 1 |

**Supplementary Table 2-3**. Amplification condition 1

| Stage | Temperature | Time |
| --- | --- | --- |
| Hold | 94℃ | 5min |
|  | 94℃ | 30sec |
| 30 Cycles | 56℃ | 30sec |
|  | 72℃ | 30sec |
| Hold | 72℃ | 5min |
| Hold | 8℃ | ∞ |

**Supplementary Table 2-4**. Reaction system 2 (for one reaction)

| Reagent | Volume (µL) |
| --- | --- |
| ddH_2_O | 9.5 |
| reaction mixture | 12.5 |
| 5µM Primers | 1 forward and 1 reverse |
| template (>10ng/ul) | 1 |

**Supplementary Table 2-5**. Amplification condition 2

| Stage | Temperature | Time |
| --- | --- | --- |
| Hold | 95℃ | 3min |
|  | 95℃ | 15sec |
| 40 Cycles | 58℃ | 15sec |
|  | 72℃ | 20sec |
| Hold | 72℃ | 7min |
| Hold | 8℃ | ∞ |

**Supplementary Table 2-6**. Reaction system 3 (for one reaction)

| Reagent | Volume (µL) |
| --- | --- |
| ddH_2_O | 5.25 |
| 2×GC Buffer | 12.5 |
| dNTP (2.5mM each) | 4 |
| Primers | 1 forward and 1 reverse |
| La Taq (5U/µL) | 0.25 |
| template (>10ng/ul) | 1 |

**Supplementary Table 2-7**. Amplification condition 3

| Stage | Temperature | Time |
| --- | --- | --- |
| Hold | 95℃ | 5min |
|  | 95℃ | 30sec |
| 10 Cycles | 68℃ | 30sec |
|  | 72℃ | 30sec |
| 35 Cycles | 94℃ | 30sec |
|  | 58℃ | 30sec |
|  | 72℃ | 30sec |
| Hold | 72℃ | 5min |
| Hold | 8℃ | ∞ |

**Supplementary Table 2-8**. Reaction system 4 and Amplification condition 4 (QF-PCR)

| **QF-PCR MIX-1** | |  | **QF-PCR MIX-2 (for one reaction)** | |  | **Reaction Condition** | | |
| --- | --- | --- | --- | --- | --- | --- | --- | --- |
| Reagent | Volume (µL) |  | Reagent | Volume (µL) |  | Stage | Temperature | Time |
| 10×Buffer | 1.0 |  | QF-PCR MIX-1 | 7.0 |  | Hold | 95℃ | 10min |
| MgSO_4_ | 0.1 |  | dNTP (2.5mM each) | 0.8 |  | 40 Cycles | 95℃ | 15sec |
| Tween20 100× | 0.1 |  | Eva Green | 0.5 |  |  | 60℃ | 1min |
| DMSO | 0.5 |  | ROX | 0.2 |  | Melt Curve | 95℃ | 15sec |
| ddH_2_O | 3.35 |  | Primer-F | 0.2 |  |  | 60℃ | 1min |
| Betaine | 2.0 |  | Primer-R | 0.2 |  |  | 95℃ | 15sec |
| Total | 7.05 |  | HS Taq | 0.1 |  |  |  |  |
|  |  |  | DNA Template | 1.0 |  |  |  |  |

**B.** In the study of Family 8, after sequence analysis, CNVs on exome level were reviewed in *TMEM38B* gene. According to the result illustrated in the following image (Supplementary Fig. 2-1), by and large it can be inferred that there is one copy loss of exon3-4 of *TMEM38B*. So, we designed a QF-PCR test to confirm that.

**Supplementary Fig. 2-1** Copy number variation in *TMEM38B* of the proband fetus (a) and a control sample (b). (Note: the copy number of exon1 is low because of the inefficiency of capturing as generally known)

**C.** In this study, two novel missense variation were detected, which associated to *FLNB*-Ala201 and *SLC26A2-*Trp98 amino acids. The biological impact prediction result of *FLNB*: c.601G>A(p.Ala201Thr) was illustrated in the main manuscript, while the structural change by *SLC26A2*: c.292T>C cannot be predicted since there has no corresponding homologous protein sequence been deconstructed. We analyzed the conservation status of *SLC26A2-*Trp98 among species via NCBI blast tool, and the result was illustrated in the image attached below.

**Supplementary Fig. 2-2** The conservation status of *SLC26A2-*Trp98 among species (by NCBI blast).
